# Supplementary material for: Dahl Salt-Resistant Rat Is Protected against Hypertension during Diet-Induced Obesity
Source: Nutrients. 2022 Sep 16;14(18):3843. doi: 10.3390/nu14183843 (PMC9506364; doi:10.3390/nu14183843)
Supplement: Supplementary file 1 [file nutrients-14-03843-s001.zip › Figure S1.pdf]

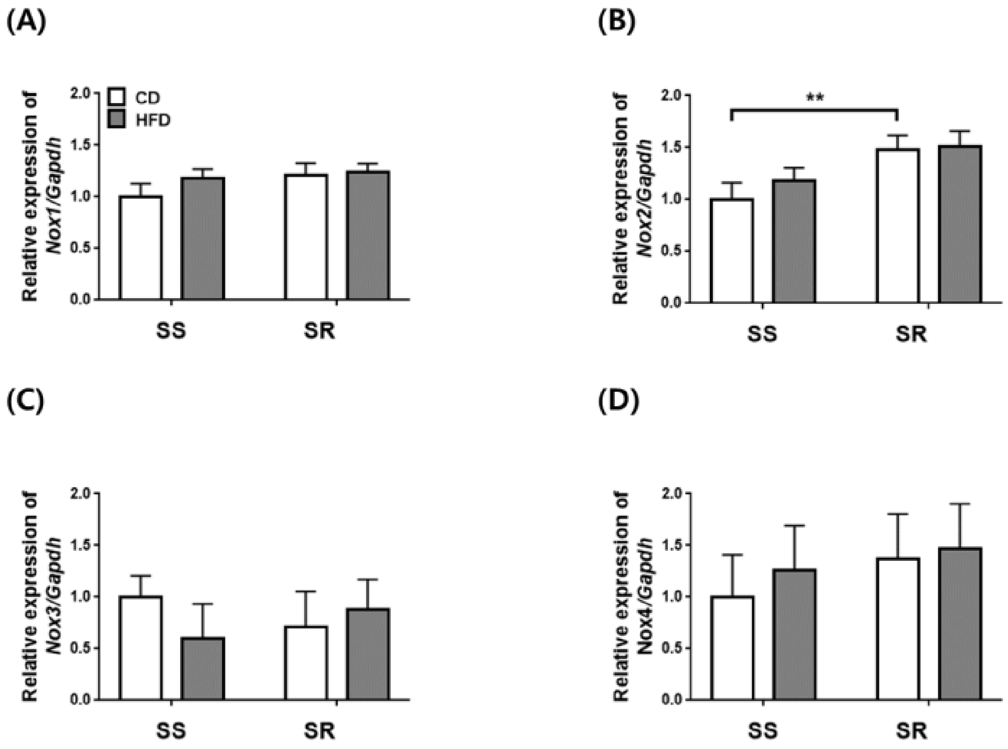

Figure S1. The effects of a high-fat diet on the expression of genes related to nicotinamide adenine dinucleotide phosphate oxidase (NADPH oxidase) in kidney from SS and SR rats. The mRNA expression of NADPH oxidase related genes such as Nox1 (A), Nox2 (B), Nox3 (C), and Nox4 (D) were measured by RT-qPCR in kidney from SS and SR rats fed a CD or HFD for 12 weeks. Graph, mean ± SEM of 6 independent experiments. Two-way analysis of variance followed by Tukey’s post hoc multiple comparisons test. **$p < 0.01$ vs. the SS CD group
