# Supplementary material for: Dahl Salt-Resistant Rat Is Protected against Hypertension during Diet-Induced Obesity
Source: Nutrients. 2022 Sep 16;14(18):3843. doi: 10.3390/nu14183843 (PMC9506364; doi:10.3390/nu14183843)
Supplement: Supplementary file 1 [file nutrients-14-03843-s001.zip › Supplementary Table S1.pdf]

Supplementary Table S1. Primers for RT-qPCR

Gene (Accession No.)	Primer sequence (5' to 3')		Tm
**RT-qPCR (for rat)**			
Ucp1	F:	TGCCTAGCAGACATCATCACC	60
(NM_012682.2)	R:	TGGCCTTCACCTTGGATCTGAA	
Pgc1α	F:	AGGACACGAGGAAAGGAAGAC	60
(NM_031347.1)	R:	GGTAGCACTGGCTTGAATCTG	
Pparγ	F:	TCAAAAGCCTGCGGAAGCCC	60
(XM_006237009.4)	R:	TGGGCGGTCTCCACTGAGAATAA	
Tmem26	F:	TTCCTGTTGCATTCCCTGGTC	57
(NM_001107623.1)	R:	GCCGGAGAAAGCCATTTGT	
Leptin	F:	TTCACACACGCAGTCGGTAT	57
(NM_013076.3)	R:	AGGTCTCGCAGGTTCTCCAG	
Ace	F:	CGGGTCGCAGAGGAATTCTT	60
(NM_012544.1)	R:	CCTGAAGTCCTTCCTGTTGTAGA	
Angiotensinogen	F:	AGAACCCCAGTGTGGAGACG	61
(XM_039086703.1)	R:	AGCCAACCTTTGAGCCTGTGCCC	
Tyrosine hydroxylase	F:	AGGGCTGCTGTCTTCCTAC	59
(XM_039101327.1)	R:	GCTGTGTCTGGGTCAAAGG	
Rarres2	F:	AAGGACTGGAAAAAGCCAGAG	58
(XM_006236415.4)	R:	TCCGGCCTAGAACTTTACCC	
Cmklr1	F:	ACCTATGCCGCTATGGAC	56
(XM_006249509.4)	R:	GACAGTGAGCAGGAAGACG	
Slc6a4	F:	GACTCCTCCCCTCTAAGCCA	60

(XM_017597042.2)	R:	CACGGAAAGAAGTGGTCGGA	
Renin	F:	GTAACTGTGGGTGGAATCATTGTG	60
(NM_012642.4)	R:	TGGGAGAGAATGTGGTCGAAGA	
Ace2	F:	TCAGAGCTGGGATGCAGAAA	60
(NM_001012006.2)	R:	GGCTCAGTCAGCATGGAGTTT	
At1ar	F:	GGAGAGGATTCGTGGCTTGAG	60
(XM_039095335.1)	R:	CTTTCTGGGAGGGTTGTGTGAT	
At1br	F:	TTGTCCACCCAATGAAGTCTCG	60
(NM_031009.2)	R:	CGCAAACTGTGATATTGGTGTTCT	
At2r	F:	CATCACCAGCAGTCTTCCTTTTG	60
(XM_006257432.4)	R:	AAAACAGTGAGACCACAACAATGT	
Mas1	F:	ACTGCCGGGCGGTCATCATC	57
(XM_039101421.1)	R:	GGTGGAGAAAAGCAAGGAGA	
Nox1	F:	AATTGGTCTCCCAAAGGAGGT	57
(XM_039108189.1)	R:	CAGGTAGAGAACAAGGTCCC	
Nox2	F:	GCATTCACACACCACTCCAC	54
(XM_006232534.4)	R:	TCCTTCCTCTCCTAAGGC	
Nox3	F:	ATCTTTATCCAGTGCCCATCC	57
(NM_001004216.1)	R:	CTTCAGTAACGCCTCTGTCCA	
Nox4	F:	AAAACCCTCCAGGCAAAGAT	57
(XM_039106434.1)	R:	TCGTCGTCGTCGTACATCTT	
Gapdh	F:	ATGACTCTACCCACGGCAAG	57
(NM_017008.4)	R:	CTGGAAGATGGTGATGGGTT	
